# Is spending time in screen-based sedentary behaviors associated with less physical activity: a cross national investigation

**DOI:** 10.1186/1479-5868-7-46

**Published:** 2010-05-21

**Authors:** Ole Melkevik, Torbjørn Torsheim, Ronald J Iannotti, Bente Wold

**Affiliations:** 1 University of Bergen, Faculty of Psychology, Christiesgate 13, N-5015 Bergen, Norway; 2 NICHD - National Institute of Child Health and Human Development 6100 Executive boulevard, 7B05 Bethesda MD20892-7510, USA

## Abstract

**Background:**

In Australia and the USA, national guidelines exist for limiting children's screen-exposure to two hours per day. This study aims to determine whether exceeding the suggested guidelines for screen-based sedentary behavior is associated with reduced levels of physical activity across different geographical regions.

**Methods:**

Data material were taken from the 2005/2006 survey of "Health Behaviour in School-aged Children (HBSC) study; A WHO cross-National Survey". Data were collected through questionnaires from 11-,13- and,15- year olds. The final sample included 200,615 adolescents from 39 different countries in Europe and North America. Gender and country stratified analyses regressed time spent in leisure-time vigorous physical activity (VPA) and days of 60 minutes moderate to vigorous physical activity (MVPA) on time spent in screen-based sedentary behaviors. To simplify interpretation, the estimates from each country were pooled using a meta-analytic procedure.

**Results:**

Exceeding 2 hrs of daily total screen-time was negatively associated with MVPA for both boys and girls, and with VPA for girls. When investigating the different types of screen-based behaviors separately, exceeding 2 hrs daily of TV viewing was associated with less MVPA for both boys and girls and less VPA for girls. Gaming was associated with less MVPA and VPA for boys, and non-gaming computer use was associated with higher levels of VPA for both genders. Stronger negative associations between physical activity and screen-based sedentary behaviors were found in countries where mean levels of physical activity were relatively high. The association between physical activity and sedentary behavior was not significantly associated with national levels of screen-based sedentary behaviors.

**Conclusions:**

The displacement mechanism does not appear to be universal across countries. On a national level, negative associations between physical activity and screen-based sedentary behaviors are less likely to be found in countries with relatively low levels of physical activity. Consequently, national guidelines for limiting children and adolescents time in screen-based sedentary behavior may not be conducive to increasing levels of physical activity in all countries.

## Background

The growing number of attractive sedentary pursuits such as satellite TV, high-speed internet entertainment, and computer- and video-games has become a concern as some studies have identified screen-based behaviors to be positively associated with overweight, obesity and metabolic risk among children and adolescents [1-4]. One of the ways these screen-based sedentary behaviors have been hypothesized to influence health is through displacing time that could otherwise have been used for physical activity [5-7]. As children and adolescents who are physically active are less likely to suffer from numerous health risks such as adiposity [5,8], metabolic risk [3], and diabetes [8], it is of great importance to identify factors that could possibly limit a physically active lifestyle.

While several studies have investigated the relationship between physical activity and sedentary behaviors, most of these, including a large scale meta-analysis by Marshall and colleagues [9], have found time spent in sedentary behaviors to be largely uncorrelated with physical activity.

Despite limited evidence in support of a displacement mechanism, national health authorities in both USA [10] and Australia [11] have implemented national guidelines suggesting that parents should limit their children's screen-exposure to two hours per day or less. While the US guidelines, aim to limit time spent watching TV due to associations between television viewing, exposure to violence, food advertising and various health-outcomes [10], the Australian recommendations are included in their national efforts to increase physical activity among children and adolescents targeting a broader range of screen-based behaviors including both computer gaming and surfing the internet as well as TV/video viewing.

Although these guidelines are exclusively directed at the population of the respective countries, they represent a new approach which could inspire other similar measures in other countries. This is not unproblematic as some cross-national studies [12,13] have identified great variation in how much time adolescents in different parts of Europe and North America spend in different relevant leisure time activities. In light of the potential introduction of national recommendations in other countries, it would thus be important to investigate whether the types of behaviors are associated differently, or if associations are stable across countries.

In light of the reviewed literature, the current study aims to investigate whether exceeding the suggested guidelines of 2 hrs daily of screen-based sedentary behaviors is associated with less time spent in vigorous physical activity (VPA) during leisure time, or with fewer days meeting the recommended 60 minutes of daily moderate to vigorous physical activity (MVPA) among the countries participating in the 2005/06 HBSC survey.

## Methods

### Sample

The data used in this study were collected as a part of the 2005/2006 survey of "Health Behaviour in School-aged Children (HBSC) study; A WHO Cross-National Survey". Nationally representative samples were selected with school or class being the sampling unit and samples were stratified to ensure national representation. The international research protocol was followed within each country to ensure consistency in survey instruments, consent, data collection and processing procedures [14]. Surveys were administered by the class teachers, participation was voluntary, and anonymity and confidentiality of the participants were ensured.

A total of 205,939 11-, 13- and 15-year old children participated across 41 different countries. Participants from Malta and Portugal were not included in the final analysis as these countries did not include all relevant variables in their national surveys. Thus, the final sample included 200,615 adolescents from 39 different countries of which 49% were boys and 51% were girls. Response rates are not entirely comparable between the different countries due to differences in sampling procedures. Nevertheless, when taking these differences into account, both school/class and pupil response rates are estimated to exceed 70% in the majority of countries/regions [15]. More details on the HBSC study procedures can be found elsewhere [15,16].

### Measures and constructs

Time spent in screen-based sedentary behaviors were assessed through three items: "About how many hours a day do you usually watch television (including videos) in your free time?", "About how many hours a day do you play PC-games or TV-games (Playstation, Xbox, GameCube etc.) in your free time?" and "About how many hours per day do you use a PC for chatting online, surfing the internet, writing emails, homework etc. in your free time". The following nine response options were the same for all three questions: "None at all", "About half an hour a day", "About 1 hour a day", "About 2 hours a day", "About 3 hours a day", "About 4 hours a day", "About 5 hours a day", "About 6 hours a day", "About 7 or more hours a day". Vereecken and colleagues [17] evaluated test-retest reliability and relative validity (7-day TV-diary) of the item assessing hours spent watching TV and found no systematic difference was identified between test and retest (Intraclass correlations between test-retest: ICC = .76 for boys and ICC = .54 for girls. Between the test and the diary: ICC = .36 for boys and ICC = .54 for girls) The reported time adolescents usually spent watching TV was found to exceed the time reported in the TV diary by approximately one hour per day for boys, and half an hour for girls The validity of the item reflecting TV/PC gaming has, to the authors' knowledge, not been investigated.

The items assessing screen-based sedentary behaviors were dichotomized in order to test whether exceeding the recommended cutoff of 2 hrs or less daily for screen-based sedentary behaviors influenced the level of VPA or MVPA in the sample. The following items were coded 0 : "None at all", "About half an hour a day", "About 1 hour a day", "About 2 hours a day". Responses of three hours or more were considered to exceed the daily recommendations and were coded 1. In the discussion, these groups are referred to as low and high users, respectively.

In order to investigate the associations between the cumulative time spent in screen-based sedentary behaviors and physical activity, the items assessing screen-time were first recoded to be able to use them as continuous variables. "None at all" was coded 0, "About half an hour a day" was coded .5, "About 1 hour a day" was coded 1, "About 2 hours a day" was coded 2 etc. Then these variables were added into a sum-score and dichotomized, scores of 2 or less were coded 0, and scores above 2 were coded 1.

*Leisure time vigorous physical activity *(VPA) was assessed by the question "Outside school hours, how many hours per week do you usually exercise so much that you get out of breath or sweat?." The response categories were: "none", "about 30 minutes", "1 hr", "2-3 hrs", "4-6 hrs", "≥7 hrs". This item has been found to have at least partial validity as Booth et al. [18] found that adolescents who reported higher levels of VPA outperformed those with lower VPA on a running test. The responses were recoded in order to increase the interpretability of the results. "None" was coded 0, "about 30 minutes" was coded .5, "1 hr" was coded 1, "2-3 hrs" was coded 2.5, "4-6 hrs" was coded 5, and finally "≥7 hrs" was coded 7.

Moderate to vigorous physical activity (MVPA) was measured by the item "Over the past 7 days how many days were you physically active for a total of at least 60 minutes?" Prochaska and colleagues [19] found this question to be reliable and to have acceptable validity in comparison with accelerometer data.

The Family Affluence Scale (FAS II) was used to measure socioeconomic status among the participants [20]. This is a composite score consisting of four items: "Does your family own a car, van or truck?", "Do you have your own bedroom for yourself?", "During the past 12 months, how many times did you travel away on holiday with your family?", "How many computers do your family own?" Having been found to have higher associations with various health variables level than commonly used economic indicators and having demonstrated good criterion validity, the FAS II is suggested to be a valid and reliable indicator of adolescent socio-economic status [20,21].

### Statistical Analyses

All analyses were performed with STATA (version 10.01 intercooled). The descriptive tables were done with the svy, vec (linearlized) command with school-class as the level 2 sampling unit. Country and gender stratified, svy-adjusted linear regression analysis, also with school-class as level 2 sampling unit, was done to produce the estimates which would be used in the meta-analysis. Linear regression was chosen due to the large sample size and reasonably normal-distribution of the different dependent variables.

The first step in the analysis was to regress weekly hours of VPA and days of MVPA on the dummy variables indicating that the individual reported spending more than 2 hrs daily in cumulative and individual screen-based sedentary behaviors. Separate analyses were done for exceeding more than 2 hrs daily in cumulative screen-based sedentary behaviors, and for the three separate subtypes of screen-behaviors. The TV, Gaming and PC-use estimates were mutually adjusted and all regression estimates were controlled for age and family affluence effects. The coefficients in the results should be read as the predicted change in hours of weekly VPA, and in days of 60 min MVPA.

The second step in the analysis was to use coefficients and standard errors from these regression analyses by the fixed-effects meta-analytic approach, metan [22], treating each country as a separate study and using weights based upon the estimated standard errors. Pooled effects were calculated both for the entire sample as well as for the different geographical regions. The estimates for each individual country were saved and used as dependent variables in the meta-regression. While the estimates were saved per country, only regional pooled estimates are presented in the results in order to limit the size of the tables.

A meta-regression was conducted in order to investigate whether the heterogeneity in the associations beetween physical activity and screen-based sedentary behaviors could be due to national differences in mean levels of the respective behaviors. This was done by regressing the country-specific coefficients from the meta-analysis on the mean levels of the respective type of screen-based sedentary behaviors and physical activity. Estimates were adjusted for the time of year data was collected, reducing the possibility of bias due to data collection in warmer or colder months.

## Results

Table 1 shows the regions, countries principal investigators and the N for each respective country included in the current study. The table also includes the time of year the data was collected.

**Table 1:** HBSC countries included in the current study, data collection dates, PI's and N.

Country (Principal Investigator)	Data collection dates	Year	N
**North America**			
Canada (Boyce)	November 2005-June	2006	5930
USA (Iannotti)	January-May	2006	3892
**Nordic Countries**			
Denmark (Due)	February-March	2006	5741
Finland (Tynjälä)	March-May	2006	5249
Greenland (Niclasen)	March-April	2006	1366
Iceland (Bjarnason)	February-March	2006	9540
Norway (Samdal)	December 2005-January	2006	4711
Sweden (Marklund)	November-December	2005	4415
**British Isles**			
England (Morgan)	September-October	2006	4783
Ireland (Gabhainn)	April-June	2006	4894
Scotland (Currie)	February-March	2006	6190
Wales (Roberts)	January-March	2006	4409
**Central Europe**			
Austria (Dür)	February-March	2006	4848
Belgium-Flemish (Maes)	March-June	2006	4311
Belgium-French (Piette)	January-February	2006	4476
Switzerland (Kuntsche)	January-March	2006	4621
Germany (Ravens-Sieberer)	January-July	2006	7274
Luxembourg (Wagener)	February-May	2006	4387
Netherlands (Vollebergh)	September-November	2005	4278
**Baltic Countries**			
Estonia (Aasvee)	February-March	2006	4484
Lithuania (Zaborskis)	March-April	2006	5632
Latvia (Pudule)	February-April	2006	4245
**Eastern Europe**			
Bulgaria (Vasileva)	March	2006	4854
Czech Republic (Csémy)	May-June	2006	4782
Hungary (Németh)	April-May	2006	3532
Romania (Baban)	March-May	2006	4684
Russian Federation (Komkov)	March-April	2006	8231
Slovakia (Morvicova)	n.a	n.a	3882
Ukraine (Balakireva)	January-February	2006	5069
Poland (Mazur)	February-April	2006	5489
**Southern Europe**			
Spain (Rodriguez Moreno)	May	2006	8891
France (Godeau)	March-June	2006	7155
Greece (Kokkevi)	March-April	2006	3715
Croatia (Kuzman)	April	2006	4968
Israel (Harel-Fisch)	May-June	2006	5686
Italy (Cavallo)	May	2006	3951
Macedonia (Chonteva)	April-May	2006	5281
Slovenia (Jeriček)	February-March	2006	5130
Turkey (Ercan)	May-June	2006	5639
	Total N		200615

Additional file 1 shows the percentages of adolescents who report more than 2 hrs daily in cumulative screen-based sedentary behaviors, in each of the screen-based behaviors individually, and mean levels of VPA and MVPA across regions, gender and age. The results show considerable variation in all variables across regions as well as age and gender differences. Gender and age differences were all statistically significant (p < .000) as indicated by ANOVA's for differences in MVPA and VPA, and for chi-square tests for differences in the screen-based sedentary behaviors.

Regionally, the Baltic countries had the highest prevalence of high use for cumulative screen-time and all the individual screen-based behaviors, although the prevalence of high PC-use was the same in the US (22%). The region with the lowest prevalence of cumulative high-screen use was central Europe. Adolescents from the Nordic countries reported less high-use of TV and were together with the US adolescents relatively less likely to spend more than 2 hrs daily playing PC/video games.

For physical activity, adolescents from Central Europe and the Nordic countries reported the highest levels of VPA with slightly less than 3 hrs weekly. The lowest levels of VPA was reported among adolescents in Southern Europe, Eastern Europe and the Baltic countries where the mean time spent in leisure time VPA were about 2 hrs weekly. Southern and Eastern European adolescents also reported the lowest levels of MVPA with about 3.9 days weekly whereas British and Northern American adolescents reported the highest levels of weekly MVPA with an average of 4.5 days.

Across age, exceeding the 2 hr recommendation for cumulative screen-time was found to be most prevalent among 15yr-olds. 13yr-olds more commonly exceed 2 hrs daily watching TV or by playing TV/PC games while more 15yr-olds reported more than 2 hrs daily using the computer for non-gaming purposes. While mean levels of VPA appear to have a slight peak at age 13, MVPA shows a clear decline with age.

Table 2 shows the pooled regional and the total pooled effects of regressing weekly MVPA and VPA on the dummy variables indicating whether individuals exceed 2 hrs daily in both cumulative and individual screen-based sedentary behaviors. Results show significant negative associations between exceeding 2 hrs of cumulative screen-time and MVPA among both boys and girls, while associations for VPA were significant for girls only. As the MVPA coefficients refer to predicted change in days per week of 60 minutes of MVPA, the pooled estimates indicate that girls who exceed 2 hrs daily of cumulative screen use report doing 60 minutes of MVPA on average .21 days less per week (one whole day less pr 5 weeks), the overall pooled effect for boys translates into .16 days less weekly. The predicted decrease in weekly VPA for girls exceeding 2 hrs cumulative screen-use is 5.4 minutes. The strongest negative associations were found in North America where exceeding 2 hrs cumulative screen time was associated with more than half-an-hour less of VPA weekly for both boys and girls, and with a predicted decrease of one half day per week of 60 minutes MVPA for both genders.

**Table 2:** Region stratified and pooled estimates of change in weekly hrs of VPA days of 60 min MVPA regressed on exceeding 2 hrs daily total screen-time and in TV, Gaming, and PC use separately, controlled for age and family affluence

	VPA				MVPA			
	ES	95% CI	ES	95% CI	ES	95% CI	ES	95% CI
	Boy		Girl		Boy		Girl	
**Cumulative screen**								
North America	-0.58	(-0.77 to -0.38)	-0.57	(-0.72 to -0.42)	-0.54	(-0.70 to -0.38)	-0.42	(-0.55 to -0.29)
Nordic Countries	-0.51	(-0.63 to -0.38)	-0.36	(-0.45 to -0.28)	-0.47	(-0.57 to -0.37)	-0.46	(-0.54 to -0.38)
British Isles	-0.31	(-0.45 to -0.16)	-0.26	(-0.36 to -0.16)	-0.31	(-0.41 to -0.20)	-0.27	(-0.36 to -0.18)
Central Europe	0.01	(-0.08 to 0.10)	-0.21	(-0.29 to -0.14)	-0.22	(-0.29 to -0.14)	-0.21	(-0.29 to -0.14)
Baltic Countries	0.12	(-0.05 to 0.30)	-0.15	(-0.26 to -0.03)	-0.21	(-0.38 to -0.04)	-0.30	(-0.42 to -0.17)
Eastern Europe	0.13	(0.05 to 0.22)	-0.01	(-0.07 to 0.05)	-0.08	(-0.16 to 0.01)	-0.15	(-0.22 to -0.08)
South Europe	0.29	(0.22 to 0.36)	0.12	(0.07 to 0.17)	0.13	(0.06 to 0.20)	-0.02	(-0.08 to 0.04)
pooled ES	**0.03**	**(-0.01 to 0.07)**	**-0.09**	**(-0.12 to -0.06)**	**-0.16**	**(-0.20 to -0.13)**	**-0.21**	**(-0.24 to -0.18)**
**TV**								
North America	-0.39	(-0.54 to -0.24)	-0.46	(-0.58 to -0.35)	-0.29	(-0.41 to -0.16)	-0.32	(-0.44 to -0.19)
Nordic Countries	-0.27	(-0.36 to -0.19)	-0.30	(-0.37 to -0.22)	-0.18	(-0.26 to -0.10)	-0.41	(-0.48 to -0.33)
British Isles	-0.07	(-0.17 to 0.03)	-0.20	(-0.29 to -0.11)	-0.16	(-0.24 to -0.07)	-0.20	(-0.28 to -0.12)
Central Europe	0.01	(-0.07 to 0.09)	-0.22	(-0.29 to -0.15)	-0.06	(-0.13 to 0.01)	-0.18	(-0.25 to -0.10)
Baltic Countries	-0.03	(-0.14 to 0.08)	-0.09	(-0.17 to 0.00)	-0.05	(-0.14 to 0.05)	-0.16	(-0.26 to -0.07)
Eastern Europe	0.12	(0.05 to 0.18)	-0.06	(-0.11 to -0.01)	0.01	(-0.06 to 0.07)	-0.21	(-0.27 to -0.14)
South Europe	0.19	(0.13 to 0.25)	0.02	(-0.03 to 0.07)	0.10	(0.05 to 0.16)	-0.07	(-0.13 to -0.02)
Pooled ES	**0.02**	**(-0.01 to 0.05)**	**-0.12**	**(-0.14 to -0.09)**	**-0.05**	**(-0.07 to -0.02)**	**-0.20**	**(-0.22 to -0.17)**
**Game**								
North America	-0.59	(-0.79 to -0.38)	-0.14	(-0.35 to 0.06)	-0.38	(-0.54 to -0.21)	-0.35	(-0.56 to -0.15)
Nordic Countries	-0.59	(-0.74 to -0.44)	-0.31	(-0.46 to -0.16)	-0.58	(-0.66 to -0.50)	-0.06	(-0.21 to 0.08)
British Isles	-0.27	(-0.41 to -0.13)	-0.14	(-0.28 to 0.00)	-0.17	(-0.27 to -0.07)	0.03	(-0.11 to 0.16)
Central Europe	-0.17	(-0.29 to -0.05)	-0.06	(-0.18 to 0.06)	-0.22	(-0.31 to -0.14)	-0.08	(-0.2 to 0.04)
Baltic Countries	-0.05	(-0.20 to 0.10)	-0.11	(-0.26 to 0.04)	-0.17	(-0.28 to -0.06)	-0.04	(-0.20 to 0.13)
Eastern Europe	0.05	(-0.04 to 0.14)	0.08	(-0.01 to 0.17)	-0.11	(-0.18 to -0.04)	0.02	(-0.08 to 0.12)
South Europe	0.10	(0.01 to 0.19)	0.03	(-0.06 to 0.12)	0.03	(-0.04 to 0.10)	0.08	(-0.02 to 0.18)
Pooled ES	**-0.10**	**(-0.15 to -0.06)**	**-0.04**	**(-0.09 to 0.00)**	**-0.20**	**(-0.23 to -0.17)**	**-0.02**	**(-0.07 to 0.03)**
**PC**								
North America	0.20	(0.03 to 0.38)	-0.17	(-0.33 to -0.02)	0.21	(0.06 to 0.36)	0.01	(-0.12 to 0.15)
Nordic Countries	-0.09	(-0.19 to 0.01)	-0.20	(-0.29 to -0.11)	-0.10	(-0.19 to -0.01)	-0.22	(-0.31 to -0.13)
British Isles	0.07	(-0.05 to 0.20)	0.00	(-0.11 to 0.11)	-0.05	(-0.16 to 0.06)	-0.08	(-0.18 to 0.03)
Central Europe	0.10	(0.00 to 0.20)	-0.01	(-0.10 to 0.09)	0.10	(0.01 to 0.19)	-0.07	(-0.16 to 0.02)
Baltic Countries	0.16	(0.02 to 0.31)	0.16	(0.03 to 0.28)	-0.02	(-0.15 to 0.11)	-0.04	(-0.16 to 0.08)
Eastern Europe	0.19	(0.10 to 0.28)	0.19	(0.11 to 0.26)	0.11	(0.02 to 0.21)	0.12	(0.03 to 0.22)
South Europe	0.21	(0.12 to 0.30)	0.21	(0.14 to 0.28)	0.03	(-0.05 to 0.12)	0.06	(-0.02 to 0.14)
**Pooled ES**	**0.12**	**(0.08 to 0.16)**	**0.07**	**(0.03 to 0.10)**	**0.03**	**(-0.01 to 0.01)**	**-0.03**	**(-0.07 to 0.00)**

When investigating the individual screen-behaviors, more than 2 hrs daily of TV viewing showed the same pattern as cumulative screen-time, and gaming was negatively associated with both MVPA and VPA for boys. Non-gaming computer use was weakly positively associated with VPA for both genders. Regionally, there were stronger negative associations between all screen-based behaviors (except gaming for boys) in North America and the Nordic countries. In contrast, associations were generally positive or non-significant for East and Southern Europe.

The I-square statistic as presented by Higgins and Thompson [23] showed that the percentage of variation attributable to heterogeneity across the countries ranged from 16-88%, with the majority above 50%. All heterogeneity statistics were statistically significant (p < .05).

The bivariate correlations between the independent variables were all small or moderate (r = .21 to r = .39) suggesting little that the risk of bias in the results due to multicollinearity.

Table 3 shows results from meta-regression analyses aiming to investigate whether the associations between physical activity and screen-based sedentary behaviors are systematically associated with national mean levels of these behaviors. The two models: "Screen time on MVPA" and "Screen time on VPA" show the prediction of the associations between exceeding 2 hrs in cumulative screen-time in MVPA and VPA respectively. All associations between exceeding cumulative screen time and both VPA and MVPA were significantly associated with national mean levels of physical activity. Mean national levels of screen-based sedentary behaviors were not significantly associated with these estimates. This indicates that stronger negative associations between screen-based sedentary behaviors and physical activity were generally found in countries where adolescents were more physically active. Mean national levels of physical activity were also found to have significant associations with the associations of the individual screen-based behaviors.

**Table 3:** Meta-regression regressing level of displacement ^a ^on national mean levels of physical activity and screen-based sedentary behaviors.

	Boys			Girls		
	Coef	95% CI	R2	Coef	95% CI	R2
**Screen time on MVPA**						
Cumulative screen time^b^	0.06	(-0.04 to 0.15)	30.45	-0.03	(-0.09 to 0.02)	47.41
Mean MVPA^c^	-0.41	(-0.64 to-0.18)		-0.27	(-0.41 to -0.12)	
						
**Screen time on VPA**						
Cumulative screen time^b^	0.07	(-0.04 to 0.19)	36.55	0.01	(-0.07 to 0.08)	48.11
Mean VPA^d^	-0.37	(-0.57 to -0.18)		-0.25	(-.37 to -0.12)	

*Note*. (a) country estimates of the association between physical activity and screen-based sedentary behaviors; (b) refers to the mean cumulative time spent in screen-based sedentary behavior for each respective country; (c) refers to the mean number of days pr week with 60 minutes cumulative MVPA in each country; (d) refers to the mean number of hours pr week in vigorous physical activity in each country.

## Discussion

There was considerable heterogeneity across regions in the associations between exceeding 2 hrs daily in screen-based sedentary behaviors and levels of both VPA and MVPA. The variation in strength and direction of the associations between physical activity and the different screen-based sedentary behaviors supports Biddle and colleagues [24] suggesting that the various screen-based sedentary behaviors should be considered as qualitatively different behaviors. The results also show that levels of physical activity and screen-based sedentary behaviors differ between gender, age and geographical regions.

Inter-regional differences suggest that the strongest negative associations between physical activity and screen-based sedentary behaviors were found in North America and the Nordic countries. In these regions, exceeding guidelines for screen-based sedentary behavior was associated with meeting the recommended 60 minutes daily of MVPA [25] on average one half day less per week and about half an hour less of VPA weekly relative to not exceeding guidelines. In the British Isles, Central Europe and the Baltic countries associations were the more moderate suggesting that exceeding the recommendations is generally also associated with less physical activity, whereas the associations tended to be either non-significant or positive in the Southern and Eastern European countries.

Contrary to the displacement hypothesis [6], this study did not reveal a higher level of displacement in countries where adolescents spend more time in screen-based sedentary behaviors. Instead, stronger negative associations between physical activity and screen-based sedentary behaviors are found in countries where the level of physical activity is relatively high. This may be interpreted to indicate that physical inactivity is not a consequence of adolescents spending too much time in screen-based sedentary behaviors, but rather that inactive adolescents have more time spend in different sedentary pursuits. The stronger negative association between physical activity and TV for girls vs. gaming for boys may thus simply reflect that inactive girls tend to watch more TV while inactive boys tend to spend more time playing computer games.

The overall positive associations between non-gaming computer use and physical activity also suggests that using the computer for homework and other such purposes is not likely to displace time for physical activity. Consequently, the current results do not support the inclusion of this type of behavior in national recommendations.

The descriptive results suggest that boys spend more time than girls, both in screen-based sedentary behaviors and in physical activity. This is comparable to a number of other studies investigating gender differences in screen-based behaviors [26-28] and physical activity [13,29,30]. The tendency for boys to report to spend more time than girls in all these behaviors could reflect a gender related reporting bias, or simply that girls spend their leisure time doing activities not assessed by this type of survey.

Age differences show that older adolescents are more likely to be spending more than 2 hrs daily in cumulative screen-time. A similar development is evident for non-gaming computer use, while high use of both TV and gaming peak at 13yrs. This development may partially be due to a change in preference as adolescents grow older, but it is also likely that demands and/or opportunities for using computers for schoolwork among older students may be a contributing factor for the increase in this type of computer use across age.

The different types of physical activity also show different patterns across age. While the identified decrease in MVPA across age is supported by previous research [31-34], the overall mean levels of reported VPA appeared stable with a slight peak among the 13-year olds.

The prevalences of TV watching in the current study correspond well with the review done by Marshall and colleagues [28] who found an overall prevalence of 66% across the reviewed studies. The corresponding prevalence in the current results is 59% (41% exceed 2 hrs).

Even though the reported time spent in the different behaviors should be interpreted with caution as both VPA and time spent watching TV are typically over-reported [17,35] and MVPA often is under-reported [36], the current results reveal a consistent pattern of more favorable levels of both VPA and sedentary behaviors in the Northern and Central European countries compared to Southern and Eastern Europe.

There are some limitations to this study. The cross-sectional design limits the extent to which causal relationships can be assessed and the validity of self-report measures assessing the time spent in various activities is not thoroughly investigated. However, even if the item validity is judged critically, there is evidence suggesting relative validity of the items used [17,18,37].

Another limitation which may have implications for the study is that the time periods in which the different behaviors are assessed are not completely consistent. While the question about VPA specified the time frame to be "outside school-hours", questions about screen-behaviors were during "free-time". This is relevant as homework, chores, work, or other obligations are done outside school-hours, but may not be considered to be "free-time". Although these behaviors were not assessed in the HBSC survey, they should be recognized as potential confounders when interpreting the current results.

Strengths of the study include the vast sample size and the regional comparisons which have previously not been reported in the scientific literature. The methodological approach is also well suited to such a large sample as the presentation of results by regions provides both a rough overview while still providing sufficient details for interpretation.

Despite limitations, this study has several potential implications shedding new light on the relationship between physical activity and screen-based sedentary behavior. First and foremost, it shows that the guidelines for limiting screen-based sedentary behaviors as implemented in the US and Australia may not be conducive to promoting physical activity in all countries. Secondly, it highlights the importance of both local and cross-national epidemiological research. The differences across countries illustrate that single country studies would have yielded different results which in turn could lead authors to conclude differently and suggest implications and recommendations based upon biased results.

The potential health promoting benefits of reducing screen-based sedentary behaviors have been highlighted by results from smaller scale interventions. A study by Epstein and colleagues [38] found that increasing screen-based sedentary behaviors was associated with lower levels of physical activity and increased caloric intake for non-obese children. The reduction of screen-based sedentary behaviors has also been associated with positive changes in physiological measurements such as BMI and waist circumference in elementary school children [39]. However, it is uncertain whether such results could be reproduced in larger scale interventions.

Examples of future research designs that would compliment the current results could include the use of objective assessments of physical activity such as actigraphs. Validation studies or alternative or improved ways of measuring screen-based sedentary behavior would also be valuable. There is also a need for more knowledge about how both environmental and motivational factors influence children and adolescents' use of screen-based sedentary behaviors.

## Conclusions

The current study has shown that spending more than 2 hrs daily in screen-based sedentary behaviors is not consistently associated with lower levels of physical activity across genders and geographical regions. This suggests that the guidelines which are implemented in the US [10] and in Australia [11] may not be appropriate in all regions as a tool to increase levels of physical activity in the adolescent population. More importantly, the regional differences identified in this study highlight the necessity of cross national studies, suggesting that conclusions based upon local or even nationally representative studies may not be universally generalizable.

## Competing interests

The authors declare that they have no competing interests.

## Authors' contributions

All analyses as well as written sections of the manuscript were mainly done by OM. TT as the main supervisor of the project have given the first author essential feedback and advice at all stages of producing this manuscript. BW and RI have contributed significantly with comments and suggestions throughout the writing process. All authors read and approved the final manuscript.

## Supplementary Material

Additional file 1 Percentage of adolescents who exceed 2 hrs daily of screen based sedentary behaviors and mean levels of Physical activity across countries, age and gender Click here for file
