# Supplementary material for: Is spending time in screen-based sedentary behaviors associated with less physical activity: a cross national investigation
Source: Int J Behav Nutr Phys Act. 2010 May 21;7:46. doi: 10.1186/1479-5868-7-46 (PMC3224890; doi:10.1186/1479-5868-7-46)
Supplement: Additional file 1 — Percentage of adolescents who exceed 2 hrs daily of screen based sedentary behaviors and mean levels of Physical activity across countries, age and gender [file 1479-5868-7-46-S1.DOC]

| Additional file 1. Percentage of adolescents who exceed 2hrs daily of screen based sedentary behaviors and mean levels of Physical activity across countries, age and gender | | | | | | | | | | | | |
| --- | --- | --- | --- | --- | --- | --- | --- | --- | --- | --- | --- | --- |
|  | >2hr daily |  | >2hr daily |  | >2hr daily |  | >2hr daily |  | Hrs/week |  | Days |  |
|  | Total | 95%CI | TV | 95%CI | Game | 95%CI | PC | 95%CI | VPA | 95%CI | MVPA^a^ | 95% CI |
| *North America* |  |  |  |  |  |  |  |  |  |  |  |  |
| Canada | 83 % | (82.00 to 84.40) | 42 % | (40.76 to 44.00) | 20 % | (18.91 to 21.36) | 29 % | (27.34 to 30.48) | 3.10 | (3.01 to 3.17) | 4.57 | (4.49 to 4.64) |
| USA | 73 % | (71.37 to 75.20) | 39 % | (36.68 to 41.45) | 12 % | (11.16 to 13.77) | 15 % | (13.96 to 16.91) | 2.32 | (2.23 to 2.41) | 4.42 | (4.32 to 4.52) |
| **Region mean** | **79 %** | **(78.07 to 80.31)** | **41 %** | **(39.65 to 42.39)** | **16 %** | **(16.04 to 17.92)** | **22 %** | **(22.18 to 24.58)** | **2.78** | **(2.72 to 2.85)** | **4.51** | **(4.45 to 4.57)** |
| *Nordic Countries* | |  |  |  |  |  |  |  |  |  |  |  |
| Denmark | 83 % | (81.36 to 83.94) | 38 % | (36.62 to 40.06) | 20 % | (18.61 to 20.96) | 19 % | (17.28 to 20.01) | 3.53 | (3.47 to 3.61) | 4.49 | (4.42 to 4.57) |
| Finland | 77 % | (75.33 to 78.33) | 27  % | (25.86 to 28.83) | 15 % | (13.38 to 15.67) | 17 % | (15.67 to 18.32) | 3.33 | (3.26 to 3.42) | 4.58 | (4.47 to 4.69) |
| Greenland | 71 % | (67.71 to 74.90) | 35 % | (31.2 to 39.05) | 13 % | (11.14 to 15.32) | 12 % | (8.79 to 15.03) | 2.20 | (2.03 to 2.38) | 4.02 | (3.86 to 4.19) |
| Iceland | 81 % | (79.48 to 81.53) | 33 % | (31.65 to 33.76) | 15 % | (14.49 to 16.20) | 24 % | (23.31 to 25.59) | 2.89 | (2.83 to 2.95) | 4.31 | (4.26 to 4.37) |
| Norway | 78 % | (76.50 to 80.24) | 35 % | (32.99 to 36.81) | 17 % | (15.30 to 17.94) | 23 % | (21.40 to 25.13) | 3.29 | (3.19 to 3.4) | 4.07 | (3.98 to 4.16) |
| Sweden | 80 % | (77.90 to 81.26) | 30 % | (27.68 to 31.48) | 19 % | (17.16 to 20.13) | 23 % | (21.06 to 24.79) | 2.68 | (2.59 to 2.77) | 4.10 | (4.00 to 4.20) |
| **Region mean** | **80 %** | **(78.98 to 80.14)** | **33 %** | **(32.11 to 33.46)** | **16 %** | **(16.10 to 17.10)** | **20 %** | **(20.56 to 21.74)** | **3.09** | **(3.05 to 3.13)** | **4.31** | **(4.28 to 4.35)** |
| *British isles* |  |  |  |  |  |  |  |  |  |  |  |  |
| England | 78 % | (75.40 to 79.78) | 34 % | (31.40 to 36.15) | 16 % | (14.15 to 17.46) | 25 % | (23.11 to 26.98) | 2.53 | (2.44 to 2.61) | 4.26 | (4.16 to 4.36) |
| Ireland | 64 % | (62.35 to 66.09) | 36 % | (34.63 to 38.12) | 10 % | (8.81 to 10.98) | 6 % | (5.57 to 7.04) | 2.65 | (2.59 to 2.75) | 4.83 | (4.74 to 4.92) |
| Scotland | 85 % | (84.19 to 86.64) | 44 % | (42.62 to 45.79) | 22 % | (20.83 to 23.74) | 27 % | (25.62 to 28.98) | 2.50 | (2.44 to 2.56) | 4.39 | (4.31 to 4.46) |
| Wales | 86 % | (84.77 to 87.09) | 45 % | (42.79 to 46.74) | 20 % | (18.19 to 21.56) | 27 % | (25.32 to 29.00) | 2.43 | (2.36 to 2.52) | 4.36 | (4.28 to 4.45) |
| **Region mean** | **79 %** | **(77.81 to 79.71)** | **40 %** | **(39.04 to 41.02)** | **17 %** | **(16.47 to 18.05)** | **21 %** | **(20.79 to 22.62)** | **2.53** | **(2.49 to 2.57)** | **4.46** | **(4.41 to 4.50)** |
| *Central Europe* |  |  |  |  |  |  |  |  |  |  |  |  |
| Austria | 74 % | (72.24 to 76.21) | 36 % | (33.62 to 37.9) | 19 % | (17.34 to 20.59) | 18 % | (16.39 to 19.45) | 2.34 | (2.25 to 2.40) | 4.16 | (4.11 to 4.30) |
| Belgium -VLG | 80 % | (78.63 to 81.66) | 40 % | (38.28 to 42.05) | 16 % | (14.17 to 16.93) | 23 % | (21.64 to 24.88) | 2.82 | (2.74 to 2.91) | 4.20 | (3.72 to 3.87) |
| Belgium -WAL | 68 % | (66.54 to 69.86) | 31 % | (28.85 to 32.39) | 19 % | (17.42 to 20.28) | 16 % | (14.79 to 17.48) | 2.85 | (2.77 to 2.94) | 3.80 | (4.08 to 4.23) |
| Switzerland | 54 % | (52.18 to 56.43) | 18 % | (16.63 to 19.66) | 7 % | (6.243 to 8.14) | 10 % | (9.02 to 11.58) | 3.10 | (3.03 to 3.17) | 3.75 | (3.68 to 3.84) |
| Germany | 75 % | (73.14 to 76.39) | 34 % | (32.66 to 35.84) | 18 % | (16.59 to 18.93) | 18 % | (16.76 to 19.43) | 2.85 | (2.78 to 2.92) | 4.04 | (3.98 to 4.10) |
| Luxemburg | 67 % | (65.14 to 69.31) | 30 % | (28.20 to 31.63) | 15 % | (13.53 to 16.17) | 18 % | (16.09 to 19.29) | 3.02 | (2.92 to 3.10) | 3.84 | (3.76 to 3.91) |
| Netherlands | 88 % | (87.11 to 89.65) | 47 % | (45.27 to 49.55) | 24 % | (21.88 to 25.40) | 36 % | (33.50 to 38.03) | 3.67 | (3.58 to 3.76) | 4.40 | (4.31 to 4.48) |
| **Region mean** | **72 %** | **(71.74 to 73.24)** | **34 %** | **(32.86 to 34.35)** | **17 %** | **(16.15 to 17.24)** | **20 %** | **(18.91 to 20.14)** | **2.93** | **( 2.90 to 2.97)** | **4.03** | **(4.00 to 4.06)** |
| *Baltic Countries* |  |  |  |  |  |  |  |  |  |  |  |  |
| Estonia | 89 % | (87.99 to 90.17) | 51 % | (48.69 to 52.61) | 27 % | (25.71 to 29.27) | 31 % | (29.16 to 33.02) | 2.23 | (2.16 to 2.32) | 3.96 | (3.89 to 4.04) |
| Lithuania | 83 % | (82.20 to 84.36) | 56 % | (54.39 to 57.58) | 20 % | (18.76 to 21.57) | 11 % | (9.86 to 12.01) | 2.03 | (1.95 to 2.11) | 3.98 | (3.90 to 4.06) |
| Latvia | 85 % | (83.55 to 86.21) | 53 % | (50.70 to 54.59) | 22 % | (20.01 to 23.07) | 24 % | (22.03 to 25.43) | 1.91 | (1.84 to 1.99) | 4.27 | (4.18 to 4.35) |
| **Region mean** | **86 %** | **(84.91 to 86.27)** | **53 %** | **(52.30 to 54.40)** | **23 %** | **(21.91 to 23.75)** | **22 %** | **(19.91 to 22.03)** | **2.06** | **(2.01 to 2.11)** | **4.06** | **(4.01 to 4.10)** |
| *Eastern Europe* |  |  |  |  |  |  |  |  |  |  |  |  |
| Bulgaria | 89 % | (87.69 to 89.85) | 63 % | (61.61 to 64.95) | 31 % | (29.33 to 32.81) | 27 % | (25.39 to 28.91) | 1.99 | (1.92 to 2.07) | 4.02 | (3.92 to 4.12) |
| Czech Republic | 80 % | (78.60 to 81.51) | 40 % | (38.19 to 41.82) | 19 % | (17.80 to 20.50) | 14 % | (13.12 to 15.77) | 1.50 | (1.45 to 1.54) | 4.16 | (4.07 to 4.25) |
| Hungary | 71 % | (68.20 to 72.87) | 38 % | (35.69 to 40.19) | 16 % | (14.18 to 17.14) | 15 % | (13.26 to 16.58) | 2.28 | (2.19 to 2.38) | 3.91 | (3.82 to 4.01) |
| Romania | 82 % | (80.72 to 83.73) | 55 % | (53.43 to 57.51) | 33 % | (31.40 to 35.11) | 20 % | (17.89 to 21.34) | 1.72 | (1.64 to 1.79) | 3.42 | (3.31 to 3.53) |
| Russia | 77 % | (76.25 to 78.33) | 50 % | (48.27 to 50.96) | 21 % | (20.07 to 22.13) | 10 % | (9.60 to 11.22) | 1.94 | (1.88 to 2.00) | 3.29 | (3.22 to 3.36) |
| Slovakia | 87 % | (85.01 to 88.09) | 56 % | (54.47 to 58.30) | 22 % | (20.52 to 23.39) | 15 % | (12.79 to 17.00) | 2.92 | (2.84 to 3.00) | 4.87 | (4.78 to 4.97) |
| Ukraine | 77 % | (75.96 to 78.95) | 56 % | (53.98 to 57.62) | 16 % | (14.89 to 17.50) | 8 % | (7.572 to 9.41) | 1.96 | (1.88 to 2.03) | 4.12 | (4.03 to 4.20) |
| Poland | 80 % | (78.69 to 81.36) | 43 % | (41.33 to 44.49) | 22 % | (20.34 to 23.05) | 25 % | (23.62 to 27.21) | 1.93 | (1.87 to 2.00) | 4.00 | (3.93 to 4.08) |
| **Region mean** | **80 %** | **(79.72 to 80.83)** | **50 %** | **(49.55 to 0.51)** | **22 %** | **(21.93 to 23.05)** | **17 %** | **(15.95 to 17.08)** | **1.99** | **(1.96 to 2.03)** | **3.88** | **(3.84 to 3.93)** |
| *Southern Europe* | |  |  |  |  |  |  |  |  |  |  |  |
| Spain | 68 % | (66.41 to 69.13) | 34 % | (33.20 to 35.80) | 11 % | (10.54 to 12.11) | 10 % | (9.71 to 11.23) | 1.88 | (1.84 to 1.93) | 3.98 | (3.93 to 4.03) |
| France | 70 % | (68.18 to 71.10) | 34 % | (33.02 to 36.01) | 13 % | (12.24 to 14.28) | 16 % | (15.2 to 17.23) | 2.39 | (2.33 to 2.45) | 3.71 | (3.65 to 3.77) |
| Greece | 74 % | (71.85 to 75.57) | 51 % | (48.71 to 52.54) | 16 % | (14.28 to 17.16) | 7 % | (6.25 to 7.95) | 2.62 | (2.52 to 2.71) | 3.69 | (3.60 to 3.79) |
| Croatia | 78 % | (76.32 to 79.42) | 52 % | (50.07 to 53.98) | 15 % | (13.63 to 16.21) | 11 % | (10.24 to 12.46) | 1.92 | (1.84 to 1.99) | 4.36 | (4.26 to 4.46) |
| Israel | 89 % | (88.25 to 90.79) | 56 % | (54.56 to 58.45) | 34 % | (32.19 to 35.88) | 36 % | (33.91 to 37.87) | 2.16 | (2.08 to 2.25) | 3.28 | (3.17 to 3.39) |
| Italy | 71 % | (68.50 to 72.63) | 38 % | (36.11 to 40.25) | 10 % | (8.95 to 11.43) | 9 % | (7.50 to 9.84) | 2.35 | (2.27 to 2.44) | 3.72 | (3.63 to 3.82) |
| Macedonia | 73 % | (71.51 to 75.18) | 45 % | (42.49 to 46.78) | 19 % | (18.14 to 20.55) | 14 % | (13.13 to 15.38) | 1.65 | (1.55 to 1.74) | 4.17 | (4.09 to 4.25) |
| Slovenia | 75 % | (73.33 to 76.80) | 36 % | (33.85 to 37.57) | 16 % | (14.36 to 17.14) | 17 % | (15.27 to 18.25) | 2.20 | (2.12 to 2.28) | 3.94 | (3.85 to 4.03) |
| Turkey | 69 % | (67.64 to 71.61) | 42 % | (40.44 to 44.11) | 15 % | (13.88 to 16.55) | 17 % | (16.27 to 18.86) | 1.65 | (1.60 to 1.73) | 3.74 | (3.67 to 3.80) |
| **Region mean** | **74 %** | **(73.00 to 74.20)** | **43 %** | **(41.49 to 42.82)** | **17 %** | **(15.81 to 16.79)** | **15 %** | **(14.91 to 15.94)** | **2.07** | **(2.04 to 2.09)** | **3.86** | **(3.84 to 3.90)** |
| *Demographics* |  |  |  |  |  |  |  |  |  |  |  |  |
| Boy | 82 % | (81.50 to 82.15) | 43 % | (42.16 to 42.97) | 27 % | (27.04 to 27.78) | 19 % | (19.11 to 19.75) | 2.83 | (2.80 to 2.84) | 4.38 | (4.36 to 3.80) |
| Girl | 73 % | (72.77 to 73.52) | 40 % | (40.03 to 40.90) | 10 % | (9.37 to 9.84) | 18 % | (17.53 to 18.20) | 2.06 | (2.05 to 2.08) | 3.77 | (3.75 to 3.79) |
| 11 yr olds | 71 % | (70.42 to 71.52) | 37 % | (36.33 to 37.53) | 17 % | (16.66 to 17.50) | 12 % | (11.75 to 12.47) | 2.38 | (2.35 to 2.41) | 4.40 | (4.37 to 4.21) |
| 13 yr olds | 80 % | (79.71 to 80.62) | 45 % | (43.96 to 45.12) | 20 % | (19.27 to 20.12) | 19 % | (19.05 to 19.96) | 2.51 | ( 2.48 to 2.54) | 4.12 | (4.10 to 4.15) |
| 15 yr olds | 81 % | (80.23 to 81.14) | 43 % | (42.21 to 43.42) | 18 % | (17.60 to 18.44) | 24 % | (23.57 to 24.59) | 2.41 | (2.39 to 2.44) | 3.70 | (3.67 to 3.70) |
| **Total** | **77 %** | **(77.08 to 77.65)** | **41 %** | **(41.14 to 41.83)** | **18 %** | **(18.04 to 18.53)** | **19 %** | **(18.36 to 18.89)** | **2.44** | **(2.42 to 2.45)** | **4.07** | **(4.06 to 4.09)** |

Note. (a) refers to the mean number of days per week the respondents report meeting a cumulative 60 minutes of moderate to vigorous physical activity
